# Supplementary material for: USP7/Maged1-mediated H2A monoubiquitination in the paraventricular thalamus: an epigenetic mechanism involved in cocaine use disorder
Source: Nat Commun. 2023 Dec 20;14:8481. doi: 10.1038/s41467-023-44120-2 (PMC10733359; doi:10.1038/s41467-023-44120-2)
Supplement: Supplementary file 1 — Supplementary Information [file 41467_2023_44120_MOESM1_ESM.pdf]

Supplementary Information

USP7/Maged1-Mediated H2A Monoubiquitination in the Paraventricular Thalamus: An Epigenetic Mechanism Involved in Cocaine Use Disorder

Julian Cheron, Leonardo Beccari, Perrine Hagué, Romain Icick, Chloé Despontin, Teresa Carusone, Matthieu Defrance, Sagar Bhogaraju, Elena Martin-Garcia, Roberto Capellan, Rafael Maldonado, Florence Vorspan, Jérôme Bonnefont, and Alban de Kerchove d'Exaerde

Correspondence to: adekerch@ulb.ac.be

This PDF file includes:

Supplementary Results
Supplementary Figs. 1 to 15
Supplementary Tables 1 to 4
Description of Supplementary Data 1-4

Supplementary Results

RNA-seq

To assess the proportion of Vglut2 neurons in the dissected thalamus, we performed fluorescence-activated cell sorting (FACS) of Vglut2 neurons specifically expressing tdTomato, and we found that 93.1 % of sorted cells showed Vglut2 expression (Supplementary Fig. 6a). A principal component analysis separated the replicates of the microdissected thalamus data on the basis of mouse genotype and cocaine or saline treatment (Supplementary Fig.6b). In control mice, repeated cocaine injection induced upregulated expression of 252 genes and downregulated expression of 452 genes compared to the expression of these genes after saline treatment (Fig. 3b and Supplementary Fig. 6c).

A gene ontology (GO) term enrichment analysis indicated that genes with cocaine-induced downregulated expression were significantly associated with microtubule regulation, axonal projection assembly and ion transport, while genes with cocaine-induced upregulated expression were assigned to supramolecular fiber (actin) organization and apoptosis (Supplementary Fig. 6d). Thus, cocaine-dependent downregulation and upregulation of gene expression seemed to affect different, yet related, biological processes.

Only 145 genes were similarly downregulated by cocaine treatment in mice with the *Maged1*-cKO and control genotypes, and 297 genes were specifically repressed in a cocaine-dependent manner only in the *Maged1*-cKO mice (Fig. 3b). Moreover, the expression of many genes was upregulated by cocaine in *Maged1*-cKO mice (494 genes), of which the expression of 383 (77.5 %) genes was not upregulated in control mice (Fig. 3b). Thus, *Maged1* inactivation mainly led to an altered transcriptional downregulation in the thalamus after cocaine administration (Fig. 3b).

Mass spectrometry

Histones H4 and H2B and Try10 are highlighted because of their proximity to prespecified thresholds and their links with the H2A-PRC1 pathway (Fig. 3f)
PRAJA1/2, CREB1, NEDD4 (E3 ubiquitin ligase) and the PRC1 deubiquitinase USP7 are the main interactors of MAGED1 (Fig. 3l-m).

Cocaine-adaptive behaviors

To dissociate a global effect on locomotor activity rather than a specific effect on cocaine-induced sensitization of Maged1 in the PVT, we compared the locomotor activity on the first day of saline injection and the last day of cocaine injection (after reaching the ceiling level) in the rescue model, *Maged1* KO, AAV-Maged1 (PVT), and in the cKO model *Maged1*^{loxP}, AAV-Cre (PVT). While there was no spontaneous locomotor activity difference between the inactivation or the re-expression of Maged1 in the PVT in these 2 models, the sensitization was significantly stronger in the rescue group as illustrated by an interaction effect between mice group (rescue versus deletion in the PVT) and treatment (saline/cocaine) (Supplementary Fig. 13).

The primary reinforcing properties of cocaine were evaluated in *Vglut2*Cre::*Maged1*^{loxP} mice and corresponding controls using operant self-administration. Mice were first trained in 5 sessions of fixed-ratio 1 (FR1) followed by 5 sessions of FR3 (Supplementary Fig. 3b). During FR1 and FR3 sessions; no statistical differences were obtained in active nose-pokes between genotypes

(Supplementary Fig. 3a, two-way ANOVA repeated measures, genotype $F_{1,14}=1.130$, n.s.). The number of nose-pokes on the inactive lever was similarly reduced in both genotypes (Supplementary Fig. 3c). No difference was observed in active nose-pokes and number of infusions during the last two or three days of FR1 and FR3, respectively (genotype x time, $F_{9,126}=1.529$, n.s., two-way ANOVA repeated measures). Similarly, the percentage of mice reaching the criteria of operant conditioning learning was 66.7 % for WT mice and 75.0 % for *Vglut2*Cre::*Maged1*^{loxP} mice following FR1 and FR3 training (chi-squared test = 0.125, n.s.). Importantly, during the three consecutive days after the acquisition criteria of stability, discrimination and more than ten infusions, the number of active nose-poking responses was significantly higher in the *Vglut2*Cre::*Maged1*^{loxP} mice than in the control mice (Supplementary Fig. 3d).

Human data

In the clinical sample, we observed that the associations between *MAGED1* and transition from first cocaine use to CUD and between *USP7* SNPs and cocaine-induced aggression, respectively, were not confounded by sex in Cox regression models (Supplementary Tables 3 and 4). Not only these associations remained significant when sex was included as a potential confounder in the model, but sex was not associated with the clinical outcomes per se. We also investigated associations between biological sex and both cocaine-induced aggression and transition from first cocaine use to CUD using bivariate analyses, showing neither was significant (cocaine-induced aggression mean =1.39 in women and 1.38 in men, median = 0 and interquartile range = 0-3 in both sexes, Mann-Whitney test, P = 0.8707; transition from first cocaine use to CUD median = 12, interquartile range = 0-60 in women and 24 months, interquartile range = 0-84 in men, log-rank test Chi2 = 1.2, P = 0.2).

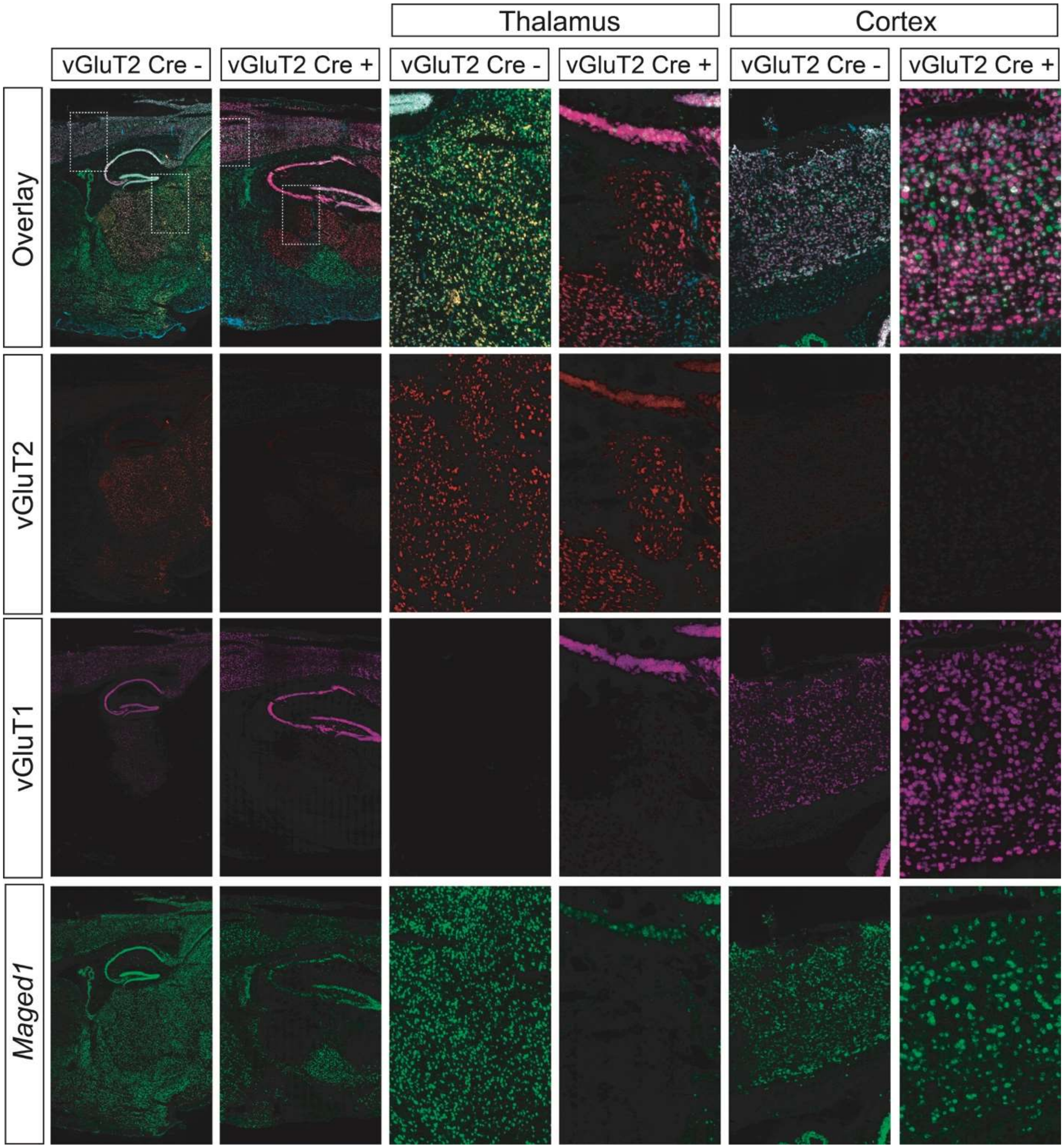

Supplementary Figure 1. Multiplexed fluorescent in situ hybridization of *Maged1*, *Vglut1* and *Vglut2*.

Multiplexed fluorescent in situ hybridization of *Maged1*, *Vglut1* and *Vglut2* mRNAs. The first column shows a parasagittal slice of a control mouse. The third and fifth columns show a higher magnification focused on the thalamus and on the cortex, respectively. We observed that *Maged1* is ubiquitously expressed among *Vglut1* and *Vglut2* cells. It is also expressed in other types of neurons and unmarked cells. The second column shows a *Maged1*-cKO specifically in *Vglut2*

expressing neurons. The fourth and sixth columns show a higher magnification focused on the thalamus and on the cortex, respectively. We observed an absence of expression of *Maged1* in the thalamic nuclei that is *Vglut2* positive, but a normal expression in the other regions, not expressing *Vglut2* or expressing *Vglut1*.

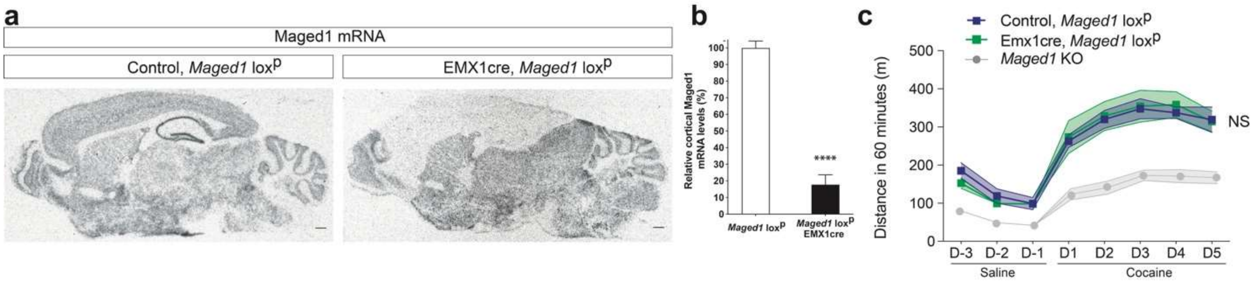

Supplementary Figure 2. *Maged1* is not necessary for cocaine-induced locomotor sensitization in glutamatergic telencephalic neurons.

a, In situ hybridization autoradiograms showing *Maged1* mRNA (scale bar: 500 µm) **b,** Quantification of the relative cortical optical densities (control, *Maged1*^{loxP}, n = 5; *Emx*1Cre:: *Maged1*^{loxP} mice, n = 5; Mann–Whitney test, P < 0.0001) **c,** Cocaine-induced locomotor sensitization (20 mg/kg, ip injection, mean ± s.e.m., control, *Maged1*^{loxP}, n = 8; *Emx1*Cre:: *Maged1*^{loxP} mice, n = 8; two-way ANOVA control versus EMX1Cre::*Maged1*^{loxP}, repeated measures, P = 0.9588; days, P < 0.0001; interaction factor (genotype x days), P = 0.9519).

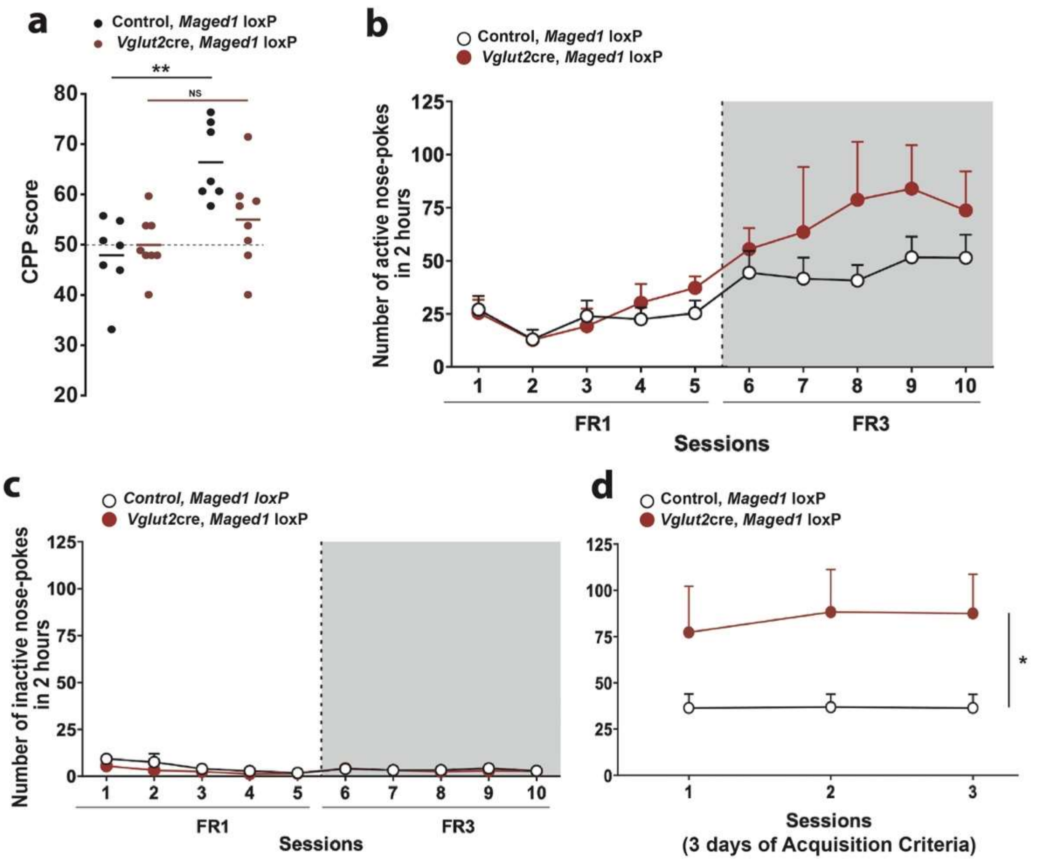

Supplementary Figure 3. Reinforcing properties of cocaine: cocaine-induced (10mg/kg, ip) conditioned place preference (CPP) and operant conditioning maintained by cocaine (0.5 mg/kg per infusion, iv) self-administration.

a, CPP scored as the percentage of time spent in the drug-paired compartment (mean ± s.e.m., n = 8 for *Vglut2*cre::*Maged1* ^{loxP} and n = 7 for control mice, two-way ANOVA, time factor, P = 0,0024, Sidak's post test, **P = 0,0028 for test versus pretest for control, Maged1^{loxP} mice and P = 0.4555 for *Vglut2*cre::*Maged1* ^{loxP} *mice*). **b,** Number of active nose-pokes during the acquisition (fixed ratio 1 (FR1) and 3 (FR3)) of self-administration, two-way ANOVA repeated measures, genotype x time, F=1.700, n.s.). **c,** Number of inactive nose-pokes during the acquisition (fixed ratio 1 (FR1) and 3 (FR3)) of self-administration, two-way ANOVA repeated measures, genotype x time, F=0.2463, n.s.). **d,** Time course of the active nose-pokes during the three days of accomplishment of acquisition criteria (mean ± S.E.M; n = 12 (control, *Maged1*^{loxP} mice), n = 4 (*Vglut2*Cre*::Maged1*^{loxP} mice), two-way ANOVA repeated measures, genotype F=7.746, P < 0.05).

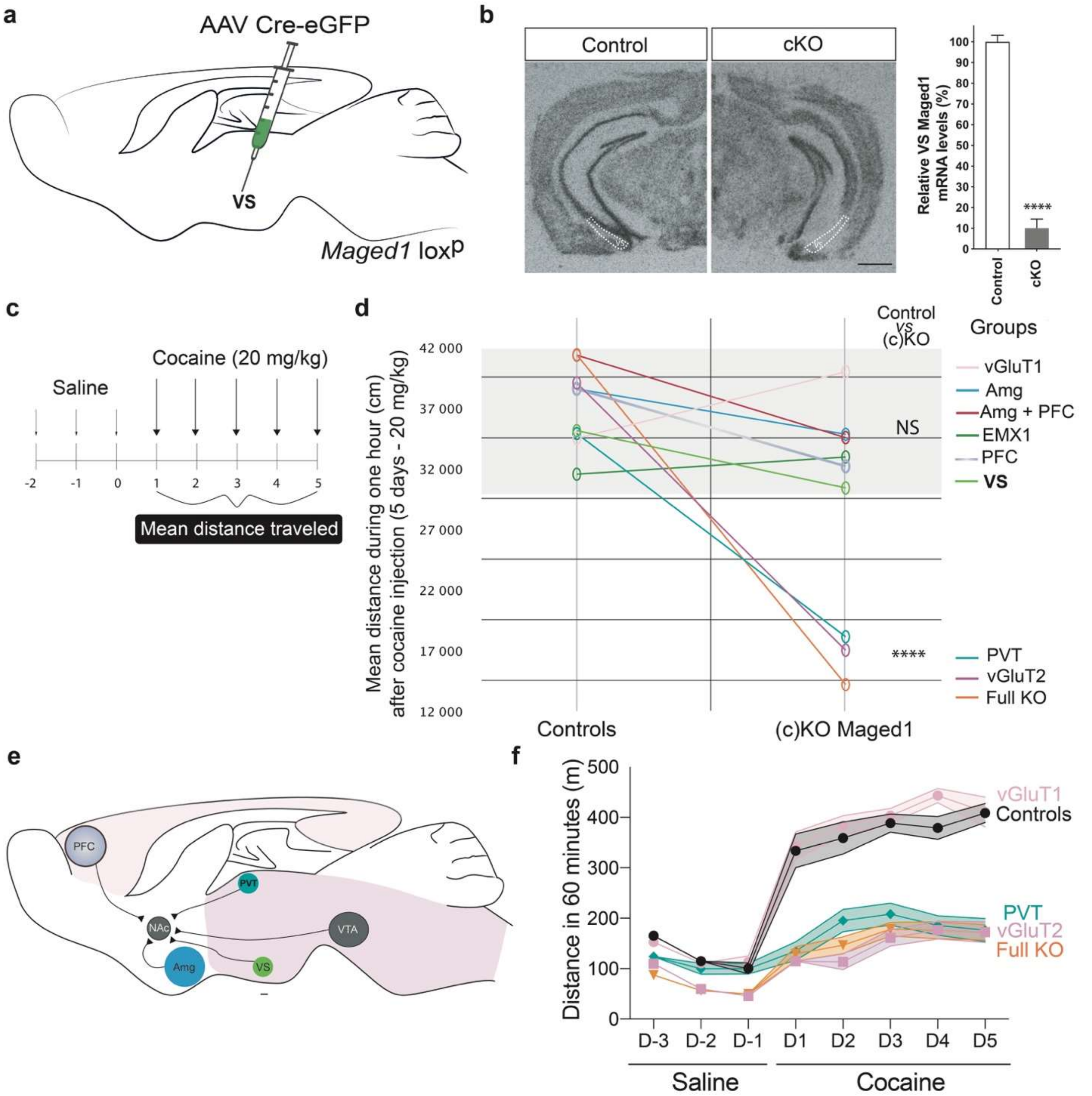

Supplementary Figure 4. Vglut2 neurons in the paraventricular thalamus are central for *Maged1* role in cocaine-induced behaviors, in contrast with other glutamatergic regions.

a,b, scheme of the experiment for ventral subiculum (VS)-specific inactivation of *Maged1* with stereotaxic injection of AAV CMV-Cre-eGFP in *Maged1*^{loxP} mice (**a**), in situ hybridization autoradiograms of *Maged1* mRNA (scale bar: 1mm) and quantification of optical densities in the VS (n = 13 (control), n = 10 (*Maged1* cKO), P < 0.001, Mann-Whitney U test) (**b**). **c,d,** Scheme of the classical cocaine-induced locomotor sensitization protocol, with 3 days of saline injection followed by 5 days of cocaine injection (**c**) and mean distance traveled during the 5 days of cocaine injection in the classical sensitization protocol (**d**); segregation between (1) *Maged1* inactivation in the ventral subiculum (VS), the prefrontal cortex (PFC), the cortical and hippocampal EMX1

cells, amygdala (Amg) inactivation, double inactivation in the Amg and PFC, vGluT1 cell inactivation and (2) diencephalic *Maged1* inactivation (PVT and Vglut2) (two-way ANOVA followed by Sidak's post hoc test, groups, P < 0.0001; treatment, P < 0.0001; interaction factor, P < 0.0001). **e,** Scheme of a sagittal section of the mouse brain that represents the different regions with targeted *Maged1* inactivation. **f,** Superimposed cocaine-induced locomotor sensitization experiment showing clear superimposition of full *Maged1* KO mice with the Vglut2 mice and of the Vglut1 mice with the control mice.

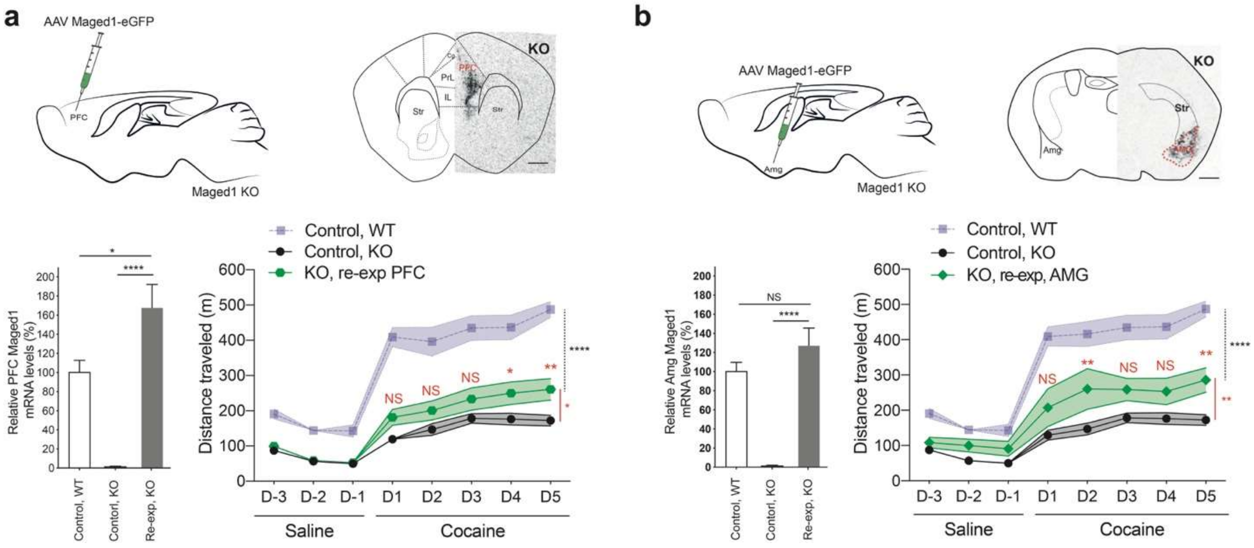

Supplementary Figure 5. Partial effect of *Maged1* re-expression in glutamatergic Vglut1/2 nuclei.

a, scheme of the experiment for PFC-specific re-expression (re-exp) of *Maged1* with stereotaxic injection of AAV CMV-*Maged1*-eGFP in *Maged1*-KO mice. In situ hybridization autoradiograms of *Maged1* mRNA (scale bar: 1mm) and quantification of optical densities in the PFC (n = 9 (control, WT), n = 11 (control, KO), n = 16 (KO, re-exp), P< 0.0001 using Kruskal-Wallis test). Cocaine induced locomotor sensitization (20 mg/kg, ip, mean ± s.e.m., n = 9 (control, WT), n = 15 (control, KO), n = 16 (KO, re-exp), control, KO versus KO, re-exp using two-way ANOVA followed by Sidak's post-test, mixed-effects model, P = 0.0268; days, P<0.0001; interaction factor (genotype x days), P = 0.0046). **b,** scheme of the experiment for Amg-specific re-exp of *Maged1* with stereotaxic injection of AAV CMV-*Maged1*-eGFP in *Maged1*-KO mice. In situ hybridization autoradiograms of *Maged1* mRNA (scale bar: 1mm) and quantification of optical densities in the Amg (n = 9 (control, WT), n = 15 (control, KO), n = 5 (KO, re-exp), P< 0.0001 using Kruskal-Wallis test). Cocaine induced locomotor sensitization (20 mg/kg, ip, mean ± s.e.m., n = 9 (control, WT), n = 15 (control, KO), n = 5 (KO, re-exp), control KO versus re-exp KO using two-way ANOVA followed by Sidak's post-test, mixed-effects model, P = 0.0020; days, P<0.0001; interaction factor (genotype x days), P = 0.0733).

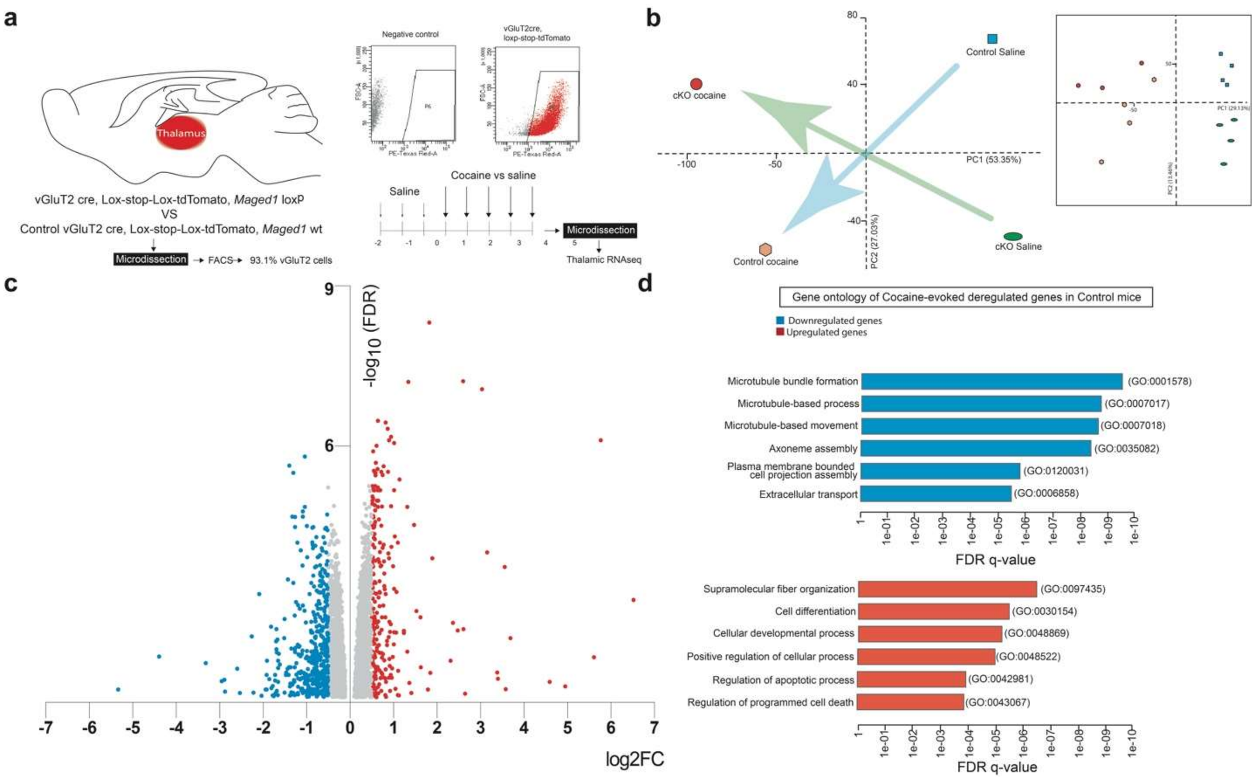

Supplementary Figure 6. Flow cytometry of the thalamus followed by transcriptomic and gene ontology analysis.

a, Scheme of the RNA-seq experiment. Flow cytometry showed that 93.1 % of microdissected thalamic tissue consisted of Vglut2 cells (n = 6). **b,** Principal component analysis revealed four clusters referring to control and *Vglut2*Cre::*Maged1*^{loxP} (cKO) treated either with saline or cocaine during 5 consecutive days. **c,** Volcano plot showing the significantly differentially expressed genes between control saline and control cocaine mice. (452 downregulated and 252 upregulated genes, log2 fold change ≥ 0.5, false discovery rate (FDR) < 0.05). **d,** Gene Ontology analysis (biological process) showing categories with statistically significant enrichment of downregulated (top) and upregulated (bottom) genes following cocaine administration in control mice.

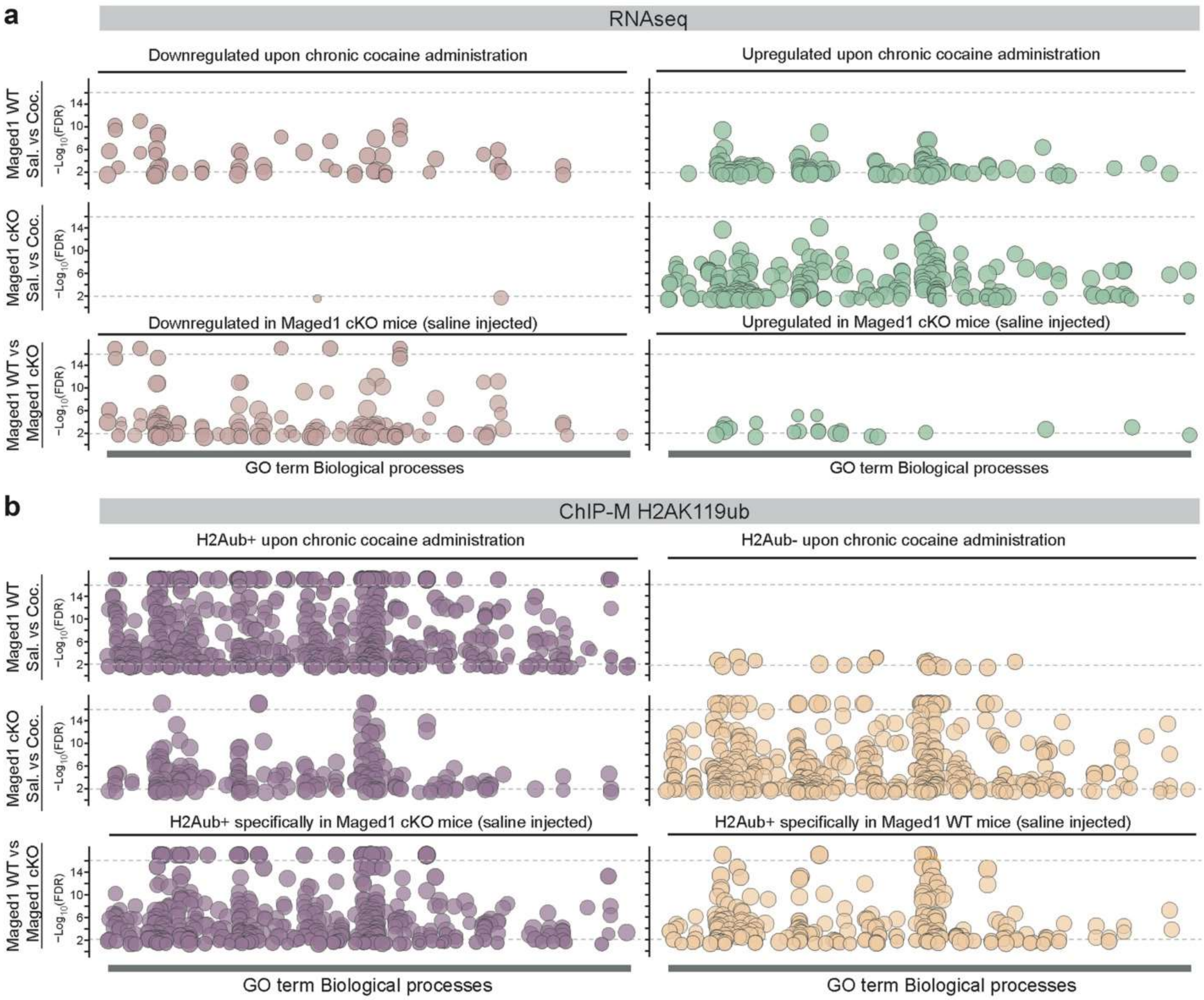

Supplementary Figure 7. Overview of the gene ontology terms.

Graphical overview of significant gene ontology (GO) terms and their –Log10 (false discovery rate, FDR) found in all the comparisons of conditions (saline versus chronic cocaine in control and *Maged1*-cKO mice and control versus *Maged1* cKO in saline-injected mice) associated to significant transcriptomic modifications (RNA-seq, **a**) and H2Aub enrichment (ChIP-M H2AK119ub, **b**). The GO terms are organized on the X-axis by their identifier number.

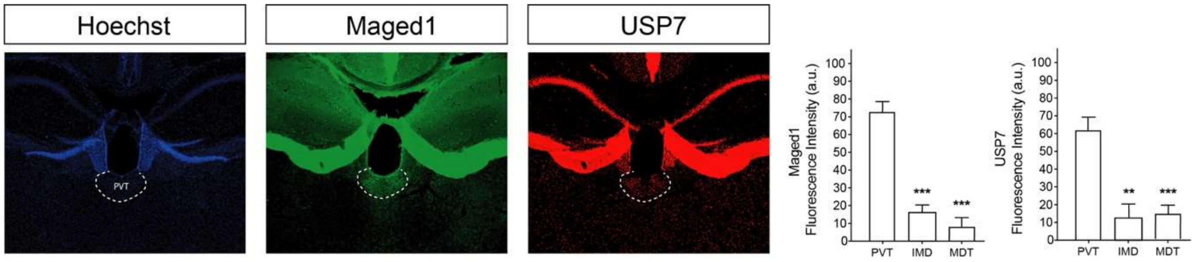

Supplementary Figure 8. Immunohistochemistry analysis of Maged1 and USP7 levels in different thalamic nuclei.

Representative images of immunohistochemistry staining for Maged1 (green), USP7 (red) and Hoechst (blue). The PVT is indicated by the dotted lines. For quantification, n = 5 mice. For Maged1: repeated-measures one-way ANOVA (*** P < 0.0001) followed by Tukey's multiple comparisons post hoc test: PVT vs IMD (*** P = 0.0001); PVT vs MDT (*** P = 0.0003); IMD vs MDT (P = 0.1552); for USP7: repeated-measure one-way ANOVA (**** P < 0.0001) followed by Tukey's multiple comparisons post hoc test: PVT vs IMD (** P = 0.0013); PVT vs MDT (*** P = 0.0006); IMD vs MDT (P = 0.7814). PVT: paraventricular nucleus of the thalamus, MDT: mediodorsal thalamic nucleus (including the medial, lateral and central parts), IMD: intermediodorsal nucleus of thalamus.

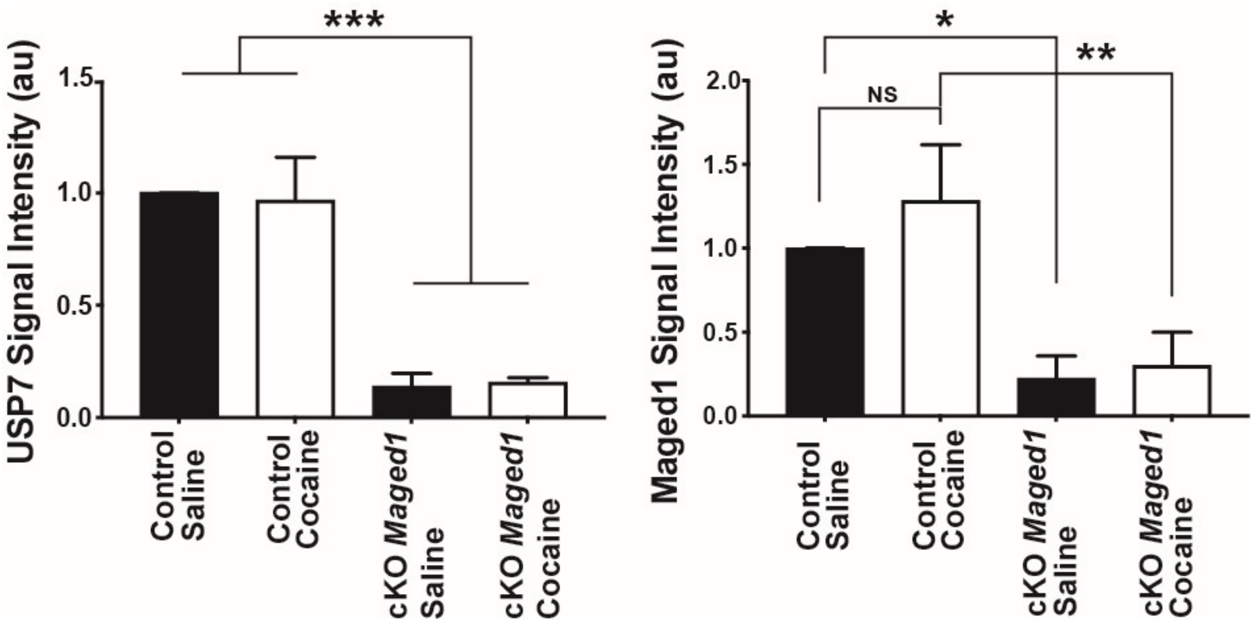

Supplementary Figure 9. Immunoprecipitation of H2Aub, western blotting quantification.

Mononucleosome IP of monoubiquitinated H2AK119 (H2Aub) and its partners with identification of Maged1 and USP7 by western blot. Quantification of USP7 (mean ± s.e.m., n = 4) (one-way ANOVA (P=0.0001) and Maged1 (mean ± s.e.m., n = 5) (one-way ANOVA (P = 0.0013) signal intensities relative to the control condition followed by Sidak's post-test *P < 0.05 **P < 0.01 ***P < 0.001).

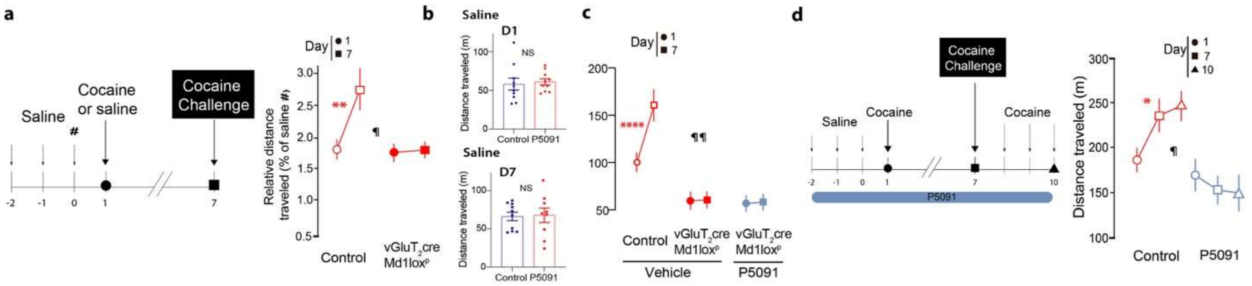

Supplementary Figure 10. Long-lasting abolition of cocaine-induced sensitization induced by USP7 inhibition, similarly to *Maged1* conditional KO, with no effect on spontaneous locomotor activity

a, Scheme of cocaine sensitization (cocaine, intraperitoneal (ip) injection, 15 mg/kg, without P5091). Cocaine-induced sensitization normalized on saline day 0 (#) was abolished after inactivation of *Maged1* in Vglut2 neurons (cKO, *Vglut2Cre*::*Maged1^{loxP}*). mean ± s.e.m., n = 9 (control), n = 8 (cKO); repeated measures two-way ANOVA: control versus cKO, P = 0.1029; days, P < 0.0301; interaction factor (genotype x days) ¶ P = 0.0370; Sidak's post test (D1 versus D7), **P = 0.0077). **b,** Locomotor activity (m) during 30 min after saline injection is not affected after 4 (top) or 10 (bottom) daily P5091 systemic administration. **c,** Cocaine-induced sensitization is abolished after Maged1 inactivation in vGluT2 cells (*Vglut2*Cre::*Maged1^{loxP}*) (vehicle and P5091 administration, i.p., 10 mg/kg (mean ± s.e.m., n = 9 (control), n = 8 (*Vglut2*Cre::*Maged1^{loxP}*, vehicle, n = 8 (*Vglut2*Cre::*Maged1^{loxP}*, P5091), repeated measures two-way ANOVA, group factor, P < 0.0001; days, P = 0.0050; interaction factor (group x days), ¶¶ P = 0.0021, Sidak's post hoc test (D1 versus D7), n.s. for *Vglut2*Cre::*Maged1^{loxP}*, vehicle and *Vglut2*Cre::*Maged1^{loxP}*, P5091, ****P<0.0001 for Control, vehicle). **d,** Scheme of the cocaine sensitization (cocaine, intraperitoneal (i.p.), 15 mg/kg) during USP7 inhibition experiment (P5091, i.p., 10 mg/kg). Cocaine-induced sensitization is abolished after systemic inhibition of USP7 (mean ± s.e.m., n = 10 (Control), n = 8 (P5091), repeated measures two-way ANOVA, vehicle versus P5091, P < 0.0001; days, P = 0.4444; interaction factor (P5091 x days), ¶ P = 0.0495, Sidak's post hoc test (D1 versus D10), * P = 0.0288).

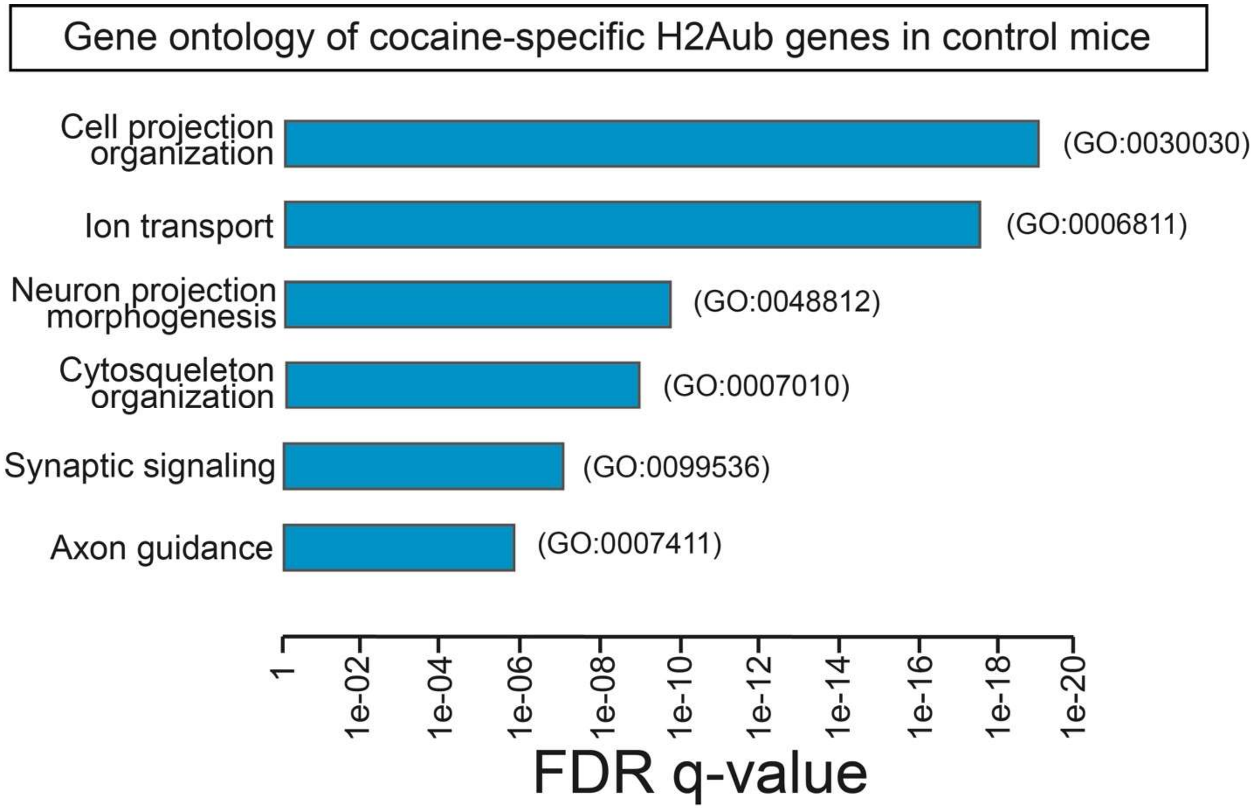

Supplementary Figure 11. Gene ontology analysis of H2Aub enrichment.
Bar plot presenting the gene ontology terms (biological process) associated with genes with H2Aub enrichment specifically in *Maged1* control mice injected with cocaine.

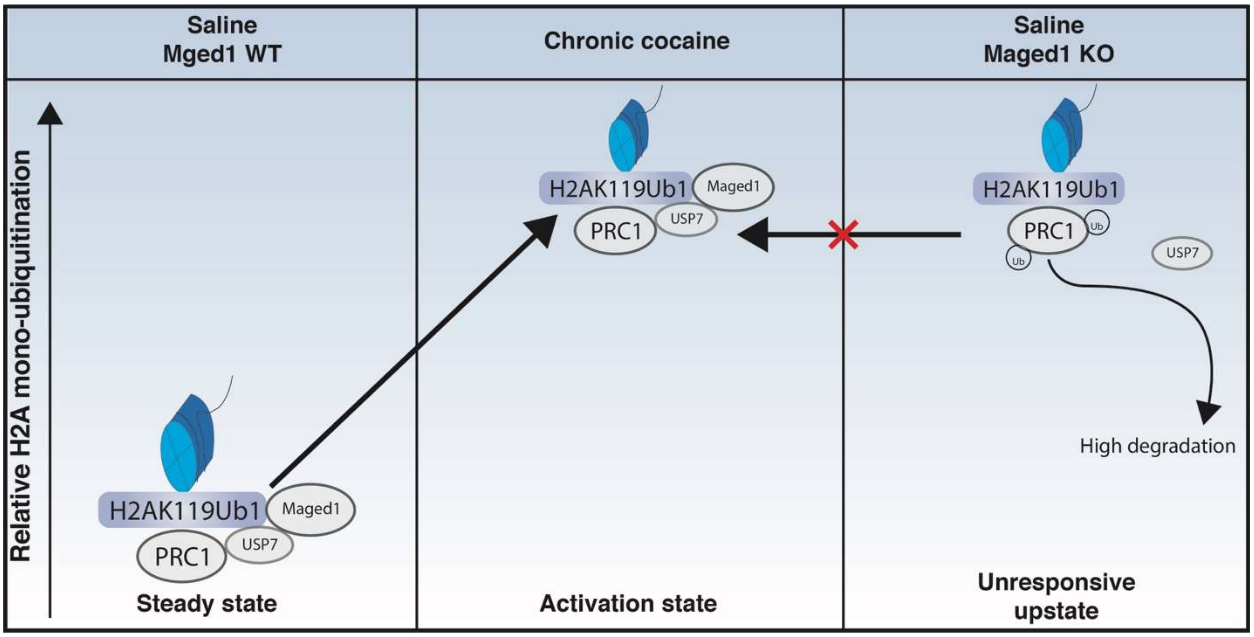

Supplementary Figure 12. Proposed mechanistic model for the role of Maged1 and USP7 in cocaine use disorder.

In this model, polycomb repressive complex 1 (PRC1), with Maged1 and USP7 are needed for cocaine-induced H2A mono-ubiquitination (H2AK119ub1). Maged1 enables the interaction between USP7 and H2AK119ub1, possibly controlling the fate of PRC1. Our results suggest that Maged1 helps, together with USP7, maintaining PRC1 in a responsive native steady state. When cocaine is administered, the complex is activated, leading to H2A mono-ubiquitination. However, in the absence of Maged1, USP7 cannot maintain PRC1 in a responsive steady state and the complex is blocked in an unresponsive upstate where upstream events (like cocaine exposure) do not modify its state.

`

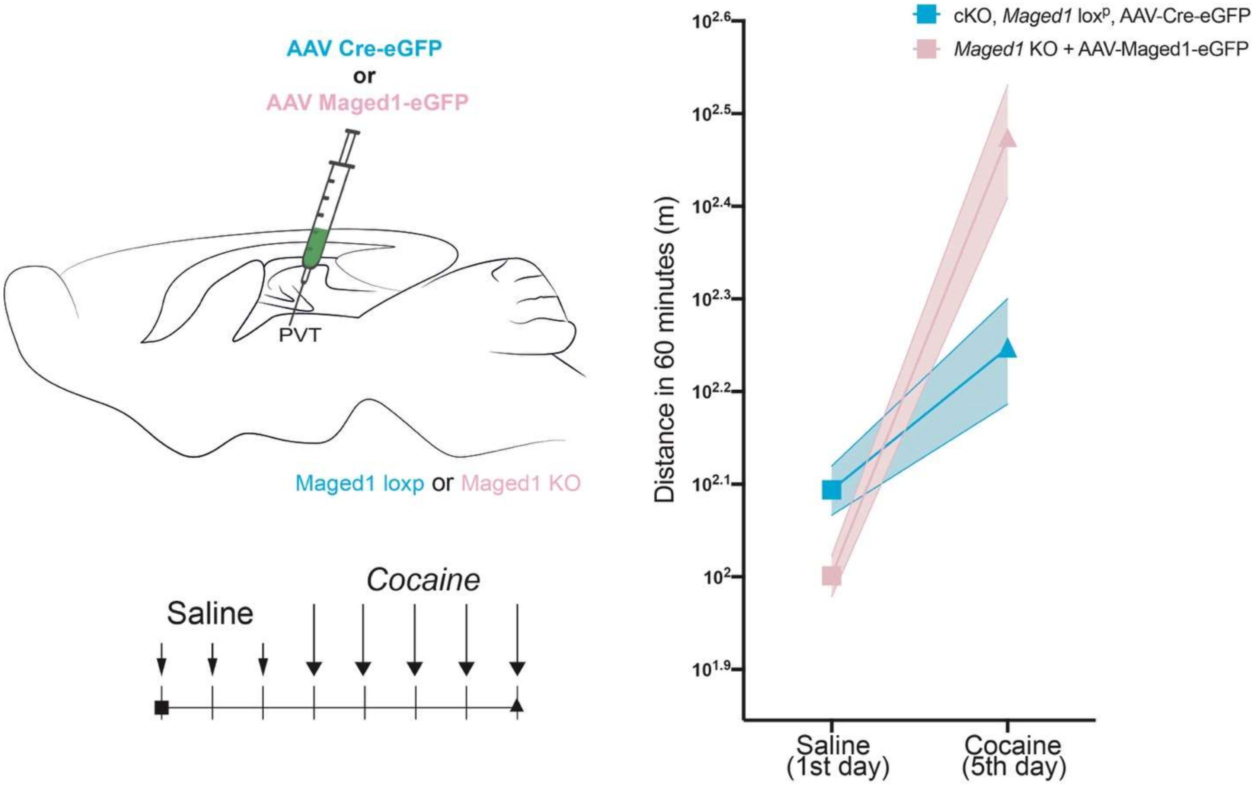

Supplementary Figure 13. Cocaine-induced locomotor sensitization is modulated by Maged1 in the PVT.

Comparison of the locomotor activity on the first day of saline injection and the last day of cocaine injection in the *Maged1*-cKO model and in the *Maged1*-KO, AAV-Maged1 rescue model (two-way ANOVA, *Maged1^{loxP}*, AAV-Cre versus *Maged1*-KO, AAV-Maged1, $P = 0.0552$; days, $P < 0.0001$; interaction factor (genotype x days), $P = 0.0037$)

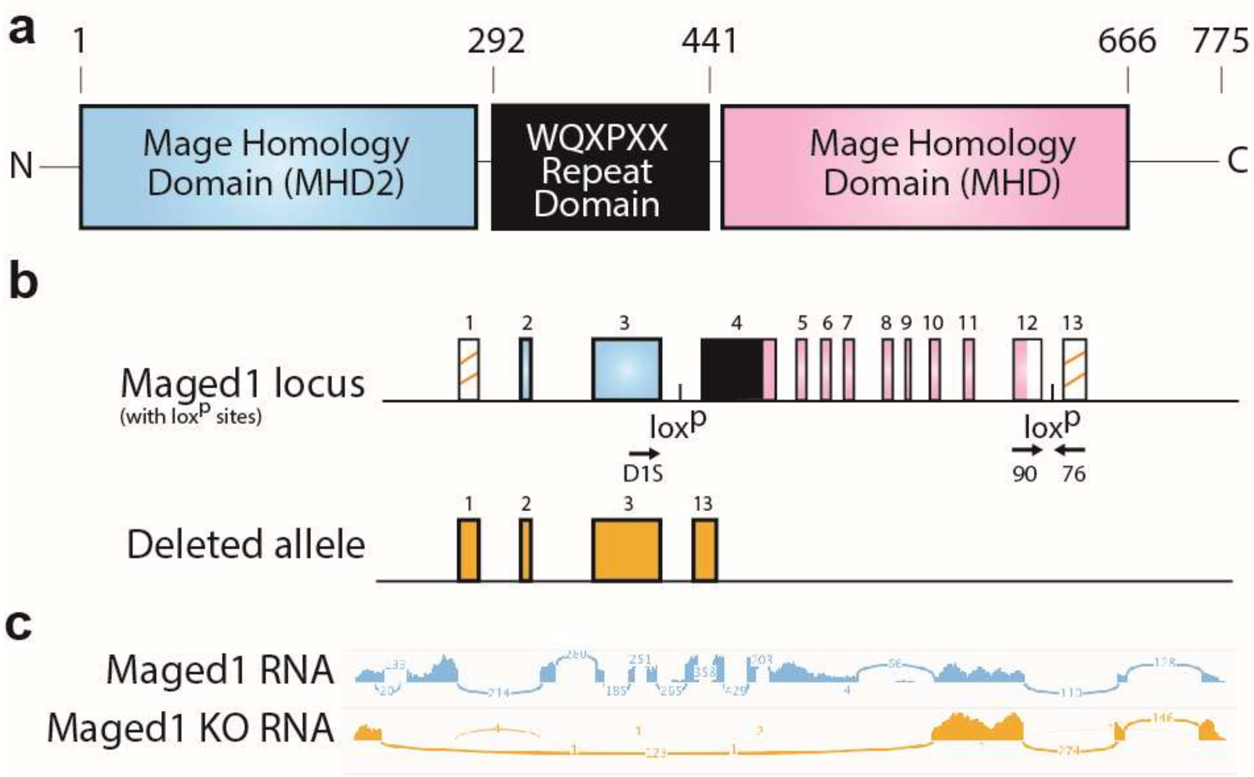

Supplementary Figure 14. Maged1 domains with their respective mRNA and DNA.
a, The Maged1 protein is composed of a conserved Mage Homology Domains (MHD) and a second MHD, MHD2 (poorly conserved between species and common to some Maged family members with no significant homology to other proteins) separated by a hexapeptide repeated domain. **b,** Schematic representation of the Maged1 locus and the knock-out allele. Boxes represent exons. The spliced part of the locus is represented with orange, with the MHD in black. Two LoxP sites were inserted into the Maged1 locus, the first one between the exons 3 and 4 and the second one between the exons 12 and 13. After recombination by a Cre recombinase, the exons 4 to 12 are deleted to obtain the (functional) Maged1 knock-out (KO) allele. Arrows represent PCR primers used for genotyping. In this work, primers 90 and 76 were used to detect wild-type and conditioned (loxP) alleles whereas the couple D1S and 76 were used to detect the KO allele[50].
c, RNA-seq mapping on the *Maged1* gene for control and KO mice.

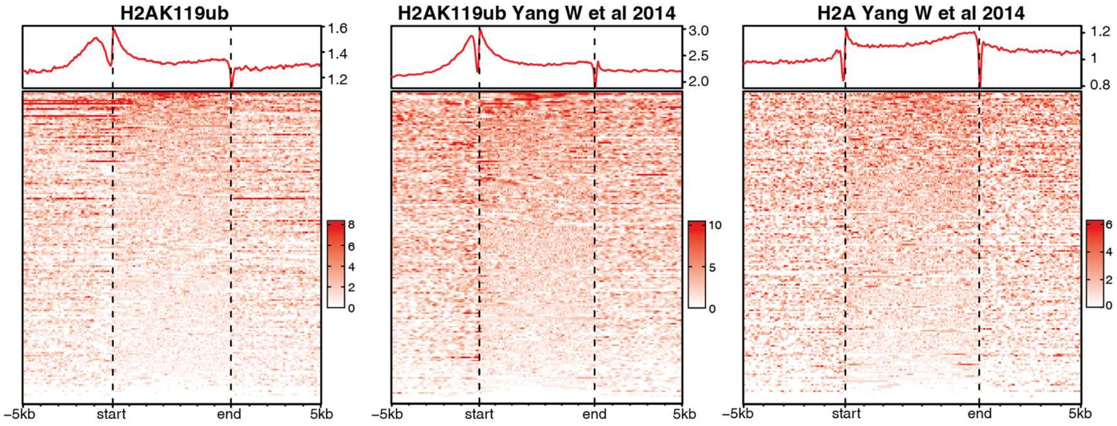

Supplementary Fig. 15. Coverage heatmap plots comparing the genome wide distribution of H2Aub over gene bodies and upstream and downstream regions reported in our study (left) with those of H2Aub and H2A form datasets of Yang W et al., 2014 (center and right).

Supplementary Table 1. Description of the clinical sample.

All SUD diagnoses are lifetime and correspond to DSM-IV TR dependence. SAPS, Scale for Assessment of Positive Symptoms – Cocaine-Induced Psychosis; IQR, interquartile range.

	N (%) or mean (SD)	**N**
Sex	82 (23%)	350
Age	38 (9)	350
Age at first cocaine use	23 (7)	338
Age at onset of cocaine use disorder	27 (8)	261
Number of cocaine uses during first year after initiation	25 (82)	203
Transition between first cocaine use and cocaine use disorder (months)	52 (72)	260
SAPS score – aggression	1.4 (1.7)	240
SAPS score – physical symptoms before cocaine use	1.3 (1.5)	240
Current tobacco smoking	304 (87%)	350
Alcohol use disorder	210 (62%)	338
Opioid use disorder	208 (61%)	340
Cannabis use disorder	217 (64%)	338
Benzodiazepine use disorder	140 (41%)	339
Number of comorbid substance use disorders (tobacco excluded)	1: 50 (14%)<br>2: 55 (16%)<br>3: 89 (26%)<br>4: 91 (26%)<br>5: 64 (18%)	350
Number of psychotropic medications in current treatment (median, IQR)	1 (0-1)	350
Number of non-psychotropic medications in current treatment (median, IQR)	1 (0-2)	350

Supplementary Table 2. Functional analysis of 54 SNPs showing significant associations (at FDR <0.05).

CHR	POS	GENE	SNP	REF	ALT	MAF_EUR	ANNOTATION	REGULOMEDB	MQTL	CADD
X	51572573	MAGED1	rs5991737	A	C	0.2386	intronic	6	1	1.456
X	51573281	MAGED1	rs1439460	T	A	0.2386	intronic	6	1	3.542
X	51581211	MAGED1	rs7061919	C	T	0.2406	intronic	2b	1	6.21
X	51581376	MAGED1	rs7056700	A	G	0.2416	intronic	3a	1	1.241
X	51581757	MAGED1	rs12853137	A	C	0.302	intronic	6	1	0.94
X	51585484	MAGED1	rs12353683	C	T	0.2435	intronic	6	1	1.714
X	51592979	MAGED1	rs7880576	T	G	0.2416	intronic	7	1	2.792
X	51601126	MAGED1	rs5991738	G	C	0.2416	intronic	7	1	5.643
X	51608351	MAGED1	rs7886851	G	A	0.2455	intronic	7	1	2.495
X	51610969	MAGED1	rs5991739	A	G	0.2435	intronic	5	1	12.47
X	51616151	MAGED1	rs6614508	T	A	0.5348	intronic	6	2	1.657
X	51621856	MAGED1	rs60919030	T	A	0.2445	intronic	6	1	2.721
X	51625979	MAGED1	rs7052927	G	A	0.2455	intronic	7	1	1.865
X	51626142	MAGED1	rs7053197	G	A	0.2455	intronic	7	1	0.8
X	51628658	MAGED1	rs12843252	A	T	0.2416	intronic	6	1	12.23
X	51628952	MAGED1	rs11795748	T	C	0.2416	intronic	7	1	2.168
X	51630805	MAGED1	rs5991707	A	T	0.2435	intronic	6	1	1.507
16	8988777	USP7	rs2304467	C	G	0.3917	3downstream,intronic	3a	2	0.695
16	8996636	USP7	rs4985062	T	C	0.4384	5upstream,non-coding intronic,intronic	5	3	4.013
16	9020056	USP7	rs9933060	C	T	0.1869	non-coding intronic,intronic	5	0	0.021
16	9023155	USP7	rs11642973	T	C	0.0775	non-coding intronic,intronic	4	1	6.523
16	9025523	USP7	rs11863279	A	G	0.1889	non-coding intronic,intronic	5	0	5.963
16	9027351	USP7	rs8045445	A	G	0.1889	non-coding intronic,intronic	6	0	12.29
16	9028645	USP7	rs12924995	A	C	0.4384	intronic,non-coding intronic	5	3	1.12
16	9029008	USP7	rs8061621	T	C	0.1889	non-coding intronic,intronic	4	0	3.903
16	9030993	USP7	rs8043595	G	C	0.1889	5upstream,intronic	5	0	1.904
16	9032015	USP7	rs7190551	G	A	0.1859	5upstream,intronic	2b	0	0.355
16	9032964	USP7	rs8059146	A	G	0.1889	intronic	5	0	4.517
16	9034716	USP7	rs11075045	A	G	0.1879	intronic	5	0	3.127
16	9034826	USP7	rs7342770	C	A	0.2147	intronic	5	2	0.095
16	9035124	USP7	rs1471435	T	C	0.3459	intronic	5	1	5.967
16	9035781	USP7	rs56410892	T	C	0.1918	intronic	4	0	0.403
16	9036452	USP7	rs12446090	T	C	0.4334	intronic	3a	4	3.297
16	9037835	USP7	rs8053087	A	T	0.1869	intronic	7	0	1.76

16	9037904	USP7	rs8049394	G	A	0.2515	intronic	5	2	1.777
16	9038117	USP7	rs12449090	T	C	0.4314	intronic	3a	4	7.254
16	9038779	USP7	rs13338372	A	G	0.1869	intronic	4	0	3.886
16	9038882	USP7	rs28651477	A	G	0.2525	intronic	5	2	2.471
16	9039334	USP7	rs11075053	T	G	0.2545	intronic	4	2	2.348
16	9040301	USP7	rs28523354	T	G	0.2545	intronic	3a	2	2.098
16	9040400	USP7	rs9932027	G	C	0.1889	intronic	4	0	2.221
16	9042631	USP7	rs2086154	A	C	0.2525	intronic	5	2	3.135
16	9043215	USP7	rs7192015	T	C	0.1918	intronic	2c	0	0.897
16	9043488	USP7	rs7192587	T	C	0.1909	5utr,intronic	3a	0	4.629
16	9043542	USP7	rs7192755	T	G	0.2535	5upstream,intronic	4	2	1.296
16	9045462	USP7	rs9926137	G	C	0.1899	5upstream,intronic	4	0	0.784
16	9047162	USP7	rs9936763	G	C	0.1859	intronic	4	0	1.398
16	9047198	USP7	rs9924441	C	T	0.2495	intronic	4	2	0.004
16	9048329	USP7	rs6498271	C	G	0.1859	intronic	3a	0	1.211
16	9049094	USP7	rs8054659	T	C	0.2535	intronic	3a	2	3.733
16	9050009	USP7	rs9939495	T	A	0.1859	intronic	4	0	3.488
16	9050331	USP7	rs12446999	C	A	0.4304	5upstream,intronic	3a	3	0.396
16	9050513	USP7	rs28727382	G	C	0.2545	5upstream,intronic	4	2	0.089
16	9051353	USP7	rs9922395	T	C	0.2545	5upstream,intronic	4	2	1.041

Supplementary Table 3. Cox regression with the transition from first cocaine use to cocaine use disorder (CUD) as the dependent variable and *MAGED1* rs7886851-A as the lead SNP. Hazard ratios (HR) and 95% confidence intervals (CI) (two-sided tests) and uncorrected *p*-values are displayed. N =127.

**Characteristic**	**HR**	**95% CI**	**p-value**
rs7886851	1.52	1.06, 2.19	0.024
Age	0.96	0.94, 0.98	<0.001
Sex	0.82	0.55, 1.23	0.3
Impulsive temper	1.00	1.00, 1.01	0.4

Supplementary Table 4. Cox regression with the transition from first cocaine use to cocaine use disorder (CUD) as the dependent variable and *MAGED1* rs6614508-A as the lead SNP. Hazard ratios (HR) and 95% confidence intervals (CI) (two-sided tests) and uncorrected *p*-values are displayed. N =127.

**Characteristic**	**HR**	**95% CI**	**p-value**
rs6614508	0.56	0.36, 0.86	0.007
Age	0.95	0.92, 0.98	<0.001
Sex	0.90	0.57, 1.43	0.7
Impulsive temper	1.00	0.99, 1.01	0.5

Description of the Supplementary Data files

Supplementary Data 1. RNAseq and ChIPmentation gene lists, Gene ontology (GO terms) and curated gene sets.

Statistical significance was set at $P < 0.05$ after correction for multiple testing using a false discovery rate procedure (Benjamini-Hochberg, BH-FDR).

Supplementary Data 2. Protein lists of the mass spectrometry results from Maged1/MAGED1 IP, Maged1/MAGED1 partners (mice and humans).

To compare protein intensities in the different conditions, student t-tests were performed. Quantified proteins and the results of the t-tests are listed. The fold change (in log2) and the adjusted statistical significance of each protein is shown.

Supplementary Data 3. Gene expression in the brain and SNPs from *MAGED1* and *USP7* with significant associations with cocaine-related phenotypes according to http://www.braineac.org/.

Supplementary Data 4. Summary statistics of associations between *MAGED1* and *USP7* SNPs and cocaine-related phenotypes.

Associations between 114 USP7 SNPs and 61 MAGED1 SNPs and seven cocaine-related phenotypes. CHR, chromosome; POS, position on chromosome (hg19 coordinates); SNP, single nucleotide polymorphism; REF, reference allele; ALT, alternate allele; PHENOTYPE, cocaine-related phenotype; N, number of cases for analysis; Z, effect magnitude; P, raw p-value; BH, FDR value after Benjamini-Hochberg correction within each phenotype (i.e. for 175 tests each time).
