# Supplementary material for: USP7/Maged1-mediated H2A monoubiquitination in the paraventricular thalamus: an epigenetic mechanism involved in cocaine use disorder
Source: Nat Commun. 2023 Dec 20;14:8481. doi: 10.1038/s41467-023-44120-2 (PMC10733359; doi:10.1038/s41467-023-44120-2)
Supplement: Supplementary file 3 — Description of Additional Supplementary Files [file 41467_2023_44120_MOESM3_ESM.pdf]

Description of additional supplementary files

File name: Supplementary Data 1

Description: RNAseq and ChIPmentation gene lists, Gene ontology (GO terms) and curated gene sets.

File name: Supplementary Data 2

Description: Protein lists of the mass spectrometry results from Maged1/MAGED1 IP, Maged1/MAGED1 partners (mice and humans).

File name: Supplementary Data 3

Description: Gene expression in the brain and SNPs from *MAGED1* and *USP7* with significant associations with cocaine-related phenotypes according to http://www.braineac.org/.

File name: Supplementary Data 4

Description: Summary statistics of associations between *MAGED1* and *USP7* SNPs and cocaine-related phenotypes.
