# Supplementary material for: USP7/Maged1-mediated H2A monoubiquitination in the paraventricular thalamus: an epigenetic mechanism involved in cocaine use disorder
Source: Nat Commun. 2023 Dec 20;14:8481. doi: 10.1038/s41467-023-44120-2 (PMC10733359; doi:10.1038/s41467-023-44120-2)
Supplement: Supplementary file 8 — Reporting Summary [file 41467_2023_44120_MOESM8_ESM.pdf]

nature portfolio

Corresponding author(s): Alban de Kerchove d'Exaerde
Last updated by author(s): 18/10/2023

Reporting Summary

Nature Portfolio wishes to improve the reproducibility of the work that we publish. This form provides structure for consistency and transparency in reporting. For further information on Nature Portfolio policies, see our Editorial Policies and the Editorial Policy Checklist.

Statistics

For all statistical analyses, confirm that the following items are present in the figure legend, table legend, main text, or Methods section.

n/a	Confirmed	
☐	☒	The exact sample size (*n*) for each experimental group/condition, given as a discrete number and unit of measurement
☐	☒	A statement on whether measurements were taken from distinct samples or whether the same sample was measured repeatedly
☐	☒	The statistical test(s) used AND whether they are one- or two-sided<br>*Only common tests should be described solely by name; describe more complex techniques in the Methods section.*
☐	☒	A description of all covariates tested
☐	☒	A description of any assumptions or corrections, such as tests of normality and adjustment for multiple comparisons
☐	☒	A full description of the statistical parameters including central tendency (e.g. means) or other basic estimates (e.g. regression coefficient) AND variation (e.g. standard deviation) or associated estimates of uncertainty (e.g. confidence intervals)
☐	☒	For null hypothesis testing, the test statistic (e.g. *F*, *t*, *r*) with confidence intervals, effect sizes, degrees of freedom and *P* value noted<br>*Give P values as exact values whenever suitable.*
☐	☒	For Bayesian analysis, information on the choice of priors and Markov chain Monte Carlo settings
☐	☒	For hierarchical and complex designs, identification of the appropriate level for tests and full reporting of outcomes
☐	☒	Estimates of effect sizes (e.g. Cohen's *d*, Pearson's *r*), indicating how they were calculated

Our web collection on statistics for biologists contains articles on many of the points above.

Software and code

Policy information about availability of computer code

Data collection	Ethovision xt 14.0 software (Noldus) was used for videotracking of mice in the open-field.
Data analysis	Locomotor state characterization and quantification was performed using Ethovision xt 14.0 software (Noldus). RNAseq mapping against the mouse reference genome (GRCm38.p4/mm10) was done using STAR 2.5.3a software. Proteins were quantified by the MaxLFQ algorithm integrated in the MaxQuant software. Further proteomic analysis was performed with the Perseus software (version 1.6.2.1) after loading the proteingroups file from MaxQuant.<br>Statistical analyses were performed using GraphPad Prism 9 software (GraphPad Software Inc.) and SPSS statistics 27 (IBM). Cell sorting was performed using a FACS Aria III cell sorter supported by the FACSDiva software (BD Biosciences).<br>The R (v4.1.2) and Rstudio (1.4.1106) softwares were used for clinical and genetic analyses and for ChIPmentation analysis. PL.INK (v. 2.0) was also used for quality control of human genetic data.

For manuscripts utilizing custom algorithms or software that are central to the research but not yet described in published literature, software must be made available to editors and reviewers. We strongly encourage code deposition in a community repository (e.g. GitHub). See the Nature Portfolio guidelines for submitting code & software for further information.

Data

Policy information about availability of data

All manuscripts must include a data availability statement. This statement should provide the following information, where applicable:

- Accession codes, unique identifiers, or web links for publicly available datasets
- A description of any restrictions on data availability
- For clinical datasets or third party data, please ensure that the statement adheres to our policy

The data supporting the findings are available within the article and its Supplementary Materials and are available from the corresponding author upon request. All RNAseq and ChIPmentation data are deposited in GEO under accession number GSE208142. The R scripts used for this study are available in GitHub upon request.

Research involving human participants, their data, or biological material

Policy information about studies with human participants or human data. See also policy information about sex, gender (identity/presentation), and sexual orientation and race, ethnicity and racism.

Reporting on sex and gender	In the part of our study involving human participants, both women and men were included.
Reporting on race, ethnicity, or other socially relevant groupings	From 592 genotyped patients, 522 were left for analysis, 351 of whom had strict Caucasian ancestry as compared to the distribution of variants in European from the 1000 genomes database.
Population characteristics	Polydrug users
Recruitment	Patients > 18 years seeking treatment for any SUD other than nicotine were consecutively recruited between April 2008 and July 2016 through two multicentric protocols.
Ethics oversight	. Both protocols and the current study were approved by the relevant Institutional Review Boards [CPP Ile-de-France IV and CEEI from the Institut de la Santé et de la Recherche Médicale (INSERM), IRB00003888 in July 2015, respectively]. All participants provided written informed consent for both the clinical and genetic assessments, and study records were continuously monitored by the hospital research administration. The research was conducted in accordance with the Helsinki Declaration as revised in 1989.

Note that full information on the approval of the study protocol must also be provided in the manuscript.

Field-specific reporting

Please select the one below that is the best fit for your research. If you are not sure, read the appropriate sections before making your selection.

☒ Life sciences ☐ Behavioural & social sciences ☐ Ecological, evolutionary & environmental sciences

For a reference copy of the document with all sections, see nature.com/documents/nr-reporting-summary-flat.pdf

Life sciences study design

All studies must disclose on these points even when the disclosure is negative.

Sample size	For all experiments, sample size was choosen according to standard practice in the field and our laboratories experience. Size was consistent with reports with similar experiments
Data exclusions	Mice from sensitization experiments were excluded if correct placement of canula or proper construct expression in the PVT (or other region of interest) were not validated.
Replication	All the experiments were replicated on 2 to 3 distinct batches. All replicatopn attempts were succesfull.
Randomization	For behavior experiments, mice were randomly assigned to experimental groups through the randomization of viral constructs injected or injected drugs.
Blinding	Behavior, H2Aub/H2A ratio, Mass spectrometry, ChIPmentation and RNAsequencing analyses were performed by researchers blinded to genotype and experimental condition.

Behavioural & social sciences study design

All studies must disclose on these points even when the disclosure is negative.

Study description	*Briefly describe the study type including whether data are quantitative, qualitative, or mixed-methods (e.g. qualitative cross-sectional, quantitative experimental, mixed-methods case study).*
Research sample	*State the research sample (e.g. Harvard university undergraduates, villagers in rural India) and provide relevant demographic information (e.g. age, sex) and indicate whether the sample is representative. Provide a rationale for the study sample chosen. For studies involving existing datasets, please describe the dataset and source.*
Sampling strategy	*Describe the sampling procedure (e.g. random, snowball, stratified, convenience). Describe the statistical methods that were used to predetermine sample size OR if no sample-size calculation was performed, describe how sample sizes were chosen and provide a rationale for why these sample sizes are sufficient. For qualitative data, please indicate whether data saturation was considered, and what criteria were used to decide that no further sampling was needed.*
Data collection	*Provide details about the data collection procedure, including the instruments or devices used to record the data (e.g. pen and paper, computer, eye tracker, video or audio equipment) whether anyone was present besides the participant(s) and the researcher, and whether the researcher was blind to experimental condition and/or the study hypothesis during data collection.*
Timing	*Indicate the start and stop dates of data collection. If there is a gap between collection periods, state the dates for each sample cohort.*
Data exclusions	*If no data were excluded from the analyses, state so OR if data were excluded, provide the exact number of exclusions and the rationale behind them, indicating whether exclusion criteria were pre-established.*
Non-participation	*State how many participants dropped out/declined participation and the reason(s) given OR provide response rate OR state that no participants dropped out/declined participation.*
Randomization	*If participants were not allocated into experimental groups, state so OR describe how participants were allocated to groups, and if allocation was not random, describe how covariates were controlled.*

Ecological, evolutionary & environmental sciences study design

All studies must disclose on these points even when the disclosure is negative.

Study description	*Briefly describe the study. For quantitative data include treatment factors and interactions, design structure (e.g. factorial, nested, hierarchical), nature and number of experimental units and replicates.*
Research sample	*Describe the research sample (e.g. a group of tagged Passer domesticus, all Stenocereus thurberi within Organ Pipe Cactus National Monument), and provide a rationale for the sample choice. When relevant, describe the organism taxa, source, sex, age range and any manipulations. State what population the sample is meant to represent when applicable. For studies involving existing datasets, describe the data and its source.*
Sampling strategy	*Note the sampling procedure. Describe the statistical methods that were used to predetermine sample size OR if no sample-size calculation was performed, describe how sample sizes were chosen and provide a rationale for why these sample sizes are sufficient.*
Data collection	*Describe the data collection procedure, including who recorded the data and how.*
Timing and spatial scale	*Indicate the start and stop dates of data collection, noting the frequency and periodicity of sampling and providing a rationale for these choices. If there is a gap between collection periods, state the dates for each sample cohort. Specify the spatial scale from which the data are taken*
Data exclusions	*If no data were excluded from the analyses, state so OR if data were excluded, describe the exclusions and the rationale behind them, indicating whether exclusion criteria were pre-established.*
Reproducibility	*Describe the measures taken to verify the reproducibility of experimental findings. For each experiment, note whether any attempts to repeat the experiment failed OR state that all attempts to repeat the experiment were successful.*
Randomization	*Describe how samples/organisms/participants were allocated into groups. If allocation was not random, describe how covariates were controlled. If this is not relevant to your study, explain why.*
Blinding	*Describe the extent of blinding used during data acquisition and analysis. If blinding was not possible, describe why OR explain why blinding was not relevant to your study.*

Did the study involve field work? ☐ Yes ☐ No

Field work, collection and transport

Field conditions	*Describe the study conditions for field work, providing relevant parameters (e.g. temperature, rainfall).*
Location	*State the location of the sampling or experiment, providing relevant parameters (e.g. latitude and longitude, elevation, water depth).*
Access & import/export	*Describe the efforts you have made to access habitats and to collect and import/export your samples in a responsible manner and in compliance with local, national and international laws, noting any permits that were obtained (give the name of the issuing authority, the date of issue, and any identifying information).*
Disturbance	*Describe any disturbance caused by the study and how it was minimized.*

Reporting for specific materials, systems and methods

We require information from authors about some types of materials, experimental systems and methods used in many studies. Here, indicate whether each material, system or method listed is relevant to your study. If you are not sure if a list item applies to your research, read the appropriate section before selecting a response.

Materials & experimental systems

n/a	Involved in the study	
☐	☒	Antibodies
☐	☒	Eukaryotic cell lines
☒	☐	Palaeontology and archaeology
☐	☒	Animals and other organisms
☐	☒	Clinical data
☒	☐	Dual use research of concern
☒	☐	Plants

Methods

n/a	Involved in the study	
☐	☒	ChIP-seq
☒	☐	Flow cytometry
☒	☐	MRI-based neuroimaging

Antibodies

Antibodies used	Chicken GFP (1:2000, Abcam, ab13970), rabbit mono-ubiquitylated histone H2A (1:1500, Cell Signaling, 8240S), rabbit anti-H2A (1/1000, Merck, 07-146), goat USP7 (1/2000, Bethyl Laboratories, A303-943A), mouse mono-ubiquitylated histone H2A (1:75, Merck, 32160702), Maged1(1/300, Cosmo-Bio, BAM-74-112-EX), MAGED1 antibody (ThermoFisher, cat. no. PA5-99091)
Validation	All these antibodies were validated by the manufacturer as shown on their websites and secondly validated and calibrated in our laboratory.

Eukaryotic cell lines

Policy information about cell lines and Sex and Gender in Research

Cell line source(s)	SH-SY5Y and HEK293T cells were obtained from ATCC
Authentication	Cell lines were not authenticated
Mycoplasma contamination	No Mycoplasma contamination was found using PCR tests done regularly.
Commonly misidentified lines (See ICLAC register)	*Name any commonly misidentified cell lines used in the study and provide a rationale for their use.*

Palaeontology and Archaeology

Specimen provenance	*Provide provenance information for specimens and describe permits that were obtained for the work (including the name of the issuing authority, the date of issue, and any identifying information). Permits should encompass collection and, where applicable, export.*
Specimen deposition	*Indicate where the specimens have been deposited to permit free access by other researchers.*

Dating methods	*If new dates are provided, describe how they were obtained (e.g. collection, storage, sample pretreatment and measurement), where they were obtained (i.e. lab name), the calibration program and the protocol for quality assurance OR state that no new dates are provided.*

☐ Tick this box to confirm that the raw and calibrated dates are available in the paper or in Supplementary Information.

Ethics oversight	*Identify the organization(s) that approved or provided guidance on the study protocol, OR state that no ethical approval or guidance was required and explain why not.*

Note that full information on the approval of the study protocol must also be provided in the manuscript.

Animals and other research organisms

Policy information about studies involving animals; ARRIVE guidelines recommended for reporting animal research, and Sex and Gender in Research

Laboratory animals	Mice, C57Bl/6 - Maged1 loxP - Maged1 KO - vGlut2 Cre - 2-4 months old, males
Wild animals	The study did not involve wild animals.
Reporting on sex	All our experiments were performed in 2- to 4-month-old mice. Our experiments were conducted on hemizygous Maged1 KO males and their wild-type littermates (Maged1 WT) generated by crossing heterozygous Maged1 KO/WT females with C57Bl6J males as Maged1 KO males are deficient in sexual behavior and do not reproduce properly.
Field-collected samples	The study did not involve field-collected samples.
Ethics oversight	All methods were performed in accordance with the relevant guidelines and regulations and approved by local ethics comitees: Belgium, ethics committee ULB Pôle Santé (Protocol 695N).

Note that full information on the approval of the study protocol must also be provided in the manuscript.

Clinical data

Policy information about clinical studies
All manuscripts should comply with the ICMJE guidelines for publication of clinical research and a completed CONSORT checklist must be included with all submissions.

Clinical trial registration	NCT00894452 & NCT01569347
Study protocol	The study protocol is available at the corresponding clinical trial registration page (https://clinicaltrials.gov/ct2/show/NCT01569347?term=Vorspan&draw=2&rank=6). CONSORT checklist is not applicable to our observational study, however, our report fulfills the STREGA checklist for the report of genetic studies (see, e.g., https://doi.org/10.1371/journal.pmed.1000022), which is an extension of the STROBE guidelines.
Data collection	Participants were recruited in two waves between (i) December 2008-April 2013, total N =213 and (i) April 2012-November 2016, total N =417; from outpatient addiction centers in France, in the following cities: Paris, Lille, Clermont-Ferrand, Clichy, Ivry-sur-Seine, Saint-Etienne, Lyon and Marseille.
Outcomes	Participants for the current study were analyzed from two pooled studies registered at NCT. Assessments and data collection were strictly similar for about 80% variables and secondary objectives, which were (regarding the current study) to characterize the trajectory of substance use and substance use disorders, in particular: age at cocaine 1st experimentation, age at onset of cocaine use disorder, comorbid conditions (e.g. ADHD) - measured using standard procedures (DSM-based interviews, validated screening tools). All participants were genotyped using the same DNA array (Illumina PsychChip) and the bilogical samples underwent the same quality control procedures at the biochemical and at the biostatistical levels, to support analyses for their primary objectives, which can be summarized as the genetic basis of the variability of response in severe substance use disorders. For the second study, cocaine-related behavioral disturbances and psychotic symptoms were predefined as the main judgment criterion for the clinical study and was assessed using a standard scale developed for this purpose (see cited references in the manuscript). All the clinical and sociodemographic data were obtained using a single face-to-face interview and all the secondary analyses reported in the current study were pre-planned.

Dual use research of concern

Policy information about dual use research of concern

Hazards

Could the accidental, deliberate or reckless misuse of agents or technologies generated in the work, or the application of information presented in the manuscript, pose a threat to:

No	Yes	
☒	☐	Public health
☒	☐	National security
☒	☐	Crops and/or livestock
☒	☐	Ecosystems
☒	☐	Any other significant area

Experiments of concern

Does the work involve any of these experiments of concern:

No	Yes	
☒	☐	Demonstrate how to render a vaccine ineffective
☒	☐	Confer resistance to therapeutically useful antibiotics or antiviral agents
☒	☐	Enhance the virulence of a pathogen or render a nonpathogen virulent
☒	☐	Increase transmissibility of a pathogen
☒	☐	Alter the host range of a pathogen
☒	☐	Enable evasion of diagnostic/detection modalities
☒	☐	Enable the weaponization of a biological agent or toxin
☒	☐	Any other potentially harmful combination of experiments and agents

Plants

Seed stocks	*Report on the source of all seed stocks or other plant material used. If applicable, state the seed stock centre and catalogue number. If plant specimens were collected from the field, describe the collection location, date and sampling procedures.*
Novel plant genotypes	*Describe the methods by which all novel plant genotypes were produced. This includes those generated by transgenic approaches, gene editing, chemical/radiation-based mutagenesis and hybridization. For transgenic lines, describe the transformation method, the number of independent lines analyzed and the generation upon which experiments were performed. For gene-edited lines, describe the editor used, the endogenous sequence targeted for editing, the targeting guide RNA sequence (if applicable) and how the editor was applied.*
Authentication	*Describe any authentication procedures for each seed stock used or novel genotype generated. Describe any experiments used to assess the effect of a mutation and, where applicable, how potential secondary effects (e.g. second site T-DNA insertions, mosiacism, off-target gene editing) were examined.*

ChIP-seq

Data deposition

☒ Confirm that both raw and final processed data have been deposited in a public database such as GEO.

☒ Confirm that you have deposited or provided access to graph files (e.g. BED files) for the called peaks.

Data access links<br>*May remain private before publication.*	https://www.ncbi.nlm.nih.gov/projects/geo/query/acc.cgi?acc=GSE208142
Files in database submission	cKO_saline H2Aub_rep1_R1.fq.gz<br>cKO_saline H2Aub_rep1_R2.fq.gz<br>cKO_saline H2Aub_rep3_R1.fq.gz<br>cKO_saline H2Aub_rep3_R2.fq.gz<br>Ctrl_Cocaine_H2Aub_rep1_R1.fq.gz<br>Ctrl_Cocaine_H2Aub_rep1_R2.fq.gz<br>Ctrl_cocaine_H2Aub_rep2_R1.fq.gz<br>Ctrl_cocaine_H2Aub_rep2_R2.fq.gz<br>Ctrl_cocaine_H2Aub_rep3_R1.fq.gz<br>Ctrl_cocaine_H2Aub_rep3_R2.fq.gz<br>Ctrl_saline H2Aub_rep2_R1.fq.gz<br>Ctrl_saline H2Aub_rep2_R2.fq.gz<br>Ctrl_saline_H2Aub_rep1_R1.fq.gz<br>Ctrl_saline_H2Aub_rep1_R2.fq.gz<br>Ctrl_saline_H2Aub_rep3_R1.fq.gz<br>Ctrl_saline_H2Aub_rep3_R2.fq.gz<br>TIC_cKO_cocaine_R1.fq.gz<br>TIC_cKO_cocaine_R2.fq.gz<br>TIC_cKO_saline_R1.fq.gz

TIC_cKO_saline_R2.fq.gz
TIC_Ctrl_cocaine_R1.fq.gz
TIC_Ctrl_cocaine_R2.fq.gz
TIC_Ctrl_saline_R1.fq.gz
TIC_Ctrl_saline_R2.fq.gz
MACS_H2Aub_KO_coc_rep1.bed
MACS_H2Aub_KO_coc_rep2.bed
MACS_H2Aub_KO_sal_rep1.bed
MACS_H2Aub_KO_sal_rep2.bed
MACS_H2Aub_KO_sal_rep3.bed
MACS_H2Aub_WT_coc_r1.bed
MACS_H2Aub_WT_coc_r4.bed
MACS_H2Aub_WT_coc_r7.bed
MACS_H2Aub_WT_sal_r1.bed
MACS_H2Aub_WT_sal_r4.bed
MACS_H2Aub_WT_sal_r7.bed
cKO_saline H2Aub_rep1_R1.fq.gz. cKO_saline H2Aub_rep1_R2.fq.gz,cKO_saline H2Aub_rep3_R1.fq.gz, cKO_saline H2Aub_rep3_R2.fq.gz, Ctrl_Cocaine_H2Aub_rep1_R1.fq.gz, Ctrl_Cocaine_H2Aub_rep1_R2.fq.gz, Ctrl_cocaine_H2Aub_rep2_R1.fq.gz, Ctrl_cocaine_H2Aub_rep2_R2.fq.gz, Ctrl_cocaine_H2Aub_rep3_R1.fq.gz Ctrl_cocaine_H2Aub_rep3_R2.fq.gz, Ctrl_saline H2Aub_rep2_R1.fq.gz, Ctrl_saline H2Aub_rep2_R2.fq.gz, Ctrl_saline_H2Aub_rep1_R1.fq.gz, Ctrl_saline_H2Aub_rep1_R2.fq.gz, Ctrl_saline_H2Aub_rep3_R1.fq.gz, Ctrl_saline_H2Aub_rep3_R2.fq.gz, TIC_cKO_cocaine_R1.fq.gz, TIC_cKO_cocaine_R2.fq.gz, TIC_cKO_saline_R1.fq.gz, TIC_cKO_saline_R2.fq.gz, TIC_Ctrl_cocaine_R1.fq.gz, TIC_Ctrl_cocaine_R2.fq.gz, TIC_Ctrl_saline_R1.fq.gz, TIC_Ctrl_saline_R2.fq.gz, MACS_H2Aub_KO_coc_rep1.bed, MACS_H2Aub_KO_coc_rep2.bed, MACS_H2Aub_KO_sal_rep1.bed, MACS_H2Aub_KO_sal_rep2.bed, MACS_H2Aub_KO_sal_rep3.bed, MACS_H2Aub_WT_coc_r1.bed, MACS_H2Aub_WT_coc_r4.bed, MACS_H2Aub_WT_coc_r7.bed,MACS_H2Aub_WT_sal_r1.bedMACS_H2Aub_WT_sal_r4.bed, MACS_H2Aub_WT_sal_r7.bed

Genome browser session
(e.g. UCSC)

Provide a link to an anonymized genome browser session for "Initial submission" and "Revised version" documents only, to enable peer review. Write "no longer applicable" for "Final submission" documents.

Methodology

Replicates

2-3 independent biological replicates were performed for each experimental condition. Each replicate was produced from 2-4 pooled mouse thalami.

Sequencing depth

ChIPmentation experiments were sequence by 100bp paired end sequencing. All sample were sequenced at seq depth >10x10^6 reads/sample with >65% valid and unique pair reads

Antibodies

anti-H2AK119ub antibody (Cell Signaling, 8240S)

Peak calling parameters

MACS2 callpeak (Galaxy Version 2.1.1.20160309.3, broadpeak parameters); lower mfold=5; upper mfold=50; Band width =300; FDR treshold =0,05

Data quality

H2Aub_WT_sal_rep1: 23748
H2Aub_WT_sal_rep2: 8955
H2Aub_WT_sal_rep3: 12114
H2Aub_WT_coc_rep1: 21105
H2Aub_WT_coc_rep2: 26055
H2Aub_WT_coc_rep3: 15874
H2Aub_Maged1KO_sal_rep1: 80720
H2Aub_Maged1KO_sal_rep2: 10809
H2Aub_Maged1KO_sal_rep3: 7271
H2Aub_Maged1KO_coc_rep1: 54656
H2Aub_Maged1KO_coc_rep2: 17442

Software

ChIPmentation sequencing data were analyzed using the public Galaxy server (Afgan E et al 2018) and personalized R scripts. First, adapter sequences were trimmed from both R1 and R2 adapters using Cutadpat v3 (Martin M et al 2011; CTGTCTCTTATACACATCTCCGAGCCCACGAGAC -A CTGTCTCTTATACACATCTGACGCTGCCGACGA -q 30 -m 15), and mapped against the mouse mm10 genome assembly using Bowtie2 (v 2.3.4.1) (Langmead B et al 2012). Only alignments with a mapping quality above 30 were kept (samtools v1..2 Danecek et al. 2021).
Significantly enriched regions were called using MACS2 (v 2.1.1.20160309.3) with broadpeak calling parameters (Zhang Y et al 2008). The bam files from TIC samples were used as a control for MACS peak calling. Coverage files were normalized by the number of millions of valid paired reads of each sample.

Flow Cytometry

Plots

Confirm that:

- [ ] The axis labels state the marker and fluorochrome used (e.g. CD4-FITC).
- [ ] The axis scales are clearly visible. Include numbers along axes only for bottom left plot of group (a 'group' is an analysis of identical markers).
- [ ] All plots are contour plots with outliers or pseudocolor plots.
- [ ] A numerical value for number of cells or percentage (with statistics) is provided.

Methodology

Sample preparation	*Describe the sample preparation, detailing the biological source of the cells and any tissue processing steps used.*
Instrument	*Identify the instrument used for data collection, specifying make and model number.*
Software	*Describe the software used to collect and analyze the flow cytometry data. For custom code that has been deposited into a community repository, provide accession details.*
Cell population abundance	*Describe the abundance of the relevant cell populations within post-sort fractions, providing details on the purity of the samples and how it was determined.*
Gating strategy	*Describe the gating strategy used for all relevant experiments, specifying the preliminary FSC/SSC gates of the starting cell population, indicating where boundaries between "positive" and "negative" staining cell populations are defined.*

- [ ] Tick this box to confirm that a figure exemplifying the gating strategy is provided in the Supplementary Information.

Magnetic resonance imaging

Experimental design

Design type	*Indicate task or resting state; event-related or block design.*
Design specifications	*Specify the number of blocks, trials or experimental units per session and/or subject, and specify the length of each trial or block (if trials are blocked) and interval between trials.*
Behavioral performance measures	*State number and/or type of variables recorded (e.g. correct button press, response time) and what statistics were used to establish that the subjects were performing the task as expected (e.g. mean, range, and/or standard deviation across subjects).*

Acquisition

Imaging type(s)	*Specify: functional, structural, diffusion, perfusion.*
Field strength	*Specify in Tesla*
Sequence & imaging parameters	*Specify the pulse sequence type (gradient echo, spin echo, etc.), imaging type (EPI, spiral, etc.), field of view, matrix size, slice thickness, orientation and TE/TR/flip angle.*
Area of acquisition	*State whether a whole brain scan was used OR define the area of acquisition, describing how the region was determined.*

Diffusion MRI ☐ Used ☐ Not used

Preprocessing

Preprocessing software	*Provide detail on software version and revision number and on specific parameters (model/functions, brain extraction, segmentation, smoothing kernel size, etc.).*
Normalization	*If data were normalized/standardized, describe the approach(es): specify linear or non-linear and define image types used for transformation OR indicate that data were not normalized and explain rationale for lack of normalization.*
Normalization template	*Describe the template used for normalization/transformation, specifying subject space or group standardized space (e.g. original Talairach, MNI305, ICBM152) OR indicate that the data were not normalized.*
Noise and artifact removal	*Describe your procedure(s) for artifact and structured noise removal, specifying motion parameters, tissue signals and physiological signals (heart rate, respiration).*

Volume censoring | *Define your software and/or method and criteria for volume censoring, and state the extent of such censoring.*

Statistical modeling & inference

Model type and settings | *Specify type (mass univariate, multivariate, RSA, predictive, etc.) and describe essential details of the model at the first and second levels (e.g. fixed, random or mixed effects; drift or auto-correlation).*

Effect(s) tested | *Define precise effect in terms of the task or stimulus conditions instead of psychological concepts and indicate whether ANOVA or factorial designs were used.*

Specify type of analysis: ☐ Whole brain ☐ ROI-based ☐ Both

Statistic type for inference | *Specify voxel-wise or cluster-wise and report all relevant parameters for cluster-wise methods.*

(See Eklund et al. 2016)

Correction | *Describe the type of correction and how it is obtained for multiple comparisons (e.g. FWE, FDR, permutation or Monte Carlo).*

Models & analysis

n/a	Involved in the study
☐	☐ Functional and/or effective connectivity
☐	☐ Graph analysis
☐	☐ Multivariate modeling or predictive analysis

Functional and/or effective connectivity | *Report the measures of dependence used and the model details (e.g. Pearson correlation, partial correlation, mutual information).*

Graph analysis | *Report the dependent variable and connectivity measure, specifying weighted graph or binarized graph, subject- or group-level, and the global and/or node summaries used (e.g. clustering coefficient, efficiency, etc.).*

Multivariate modeling and predictive analysis | *Specify independent variables, features extraction and dimension reduction, model, training and evaluation metrics.*
